# Progressive Impairment of Lactate-based Gluconeogenesis in the Huntington’s Disease Mouse Model R6/2

**DOI:** 10.1371/currents.hd.019b33aae1c519e6e8b68e7cf3e7818e

**Published:** 2015-04-20

**Authors:** Signe Marie Borch Nielsen, Lis Hasholt, Anne Nørremølle, Knud Josefsen

**Affiliations:** Section of Neurogenetics, Institute of Cellular and Molecular Medicine, Faculty of Health and Medical Sciences, University of Copenhagen, Copenhagen, Denmark; The Bartholin Institute, Rigshospitalet, Copenhagen, Denmark; Section of Neurogenetics, Institute of Cellular and Molecular Medicine, Faculty of Health and Medical Sciences, University of Copenhagen, Copenhagen, Denmark; Section of Neurogenetics, Institute of Cellular and Molecular Medicine, Faculty of Health and Medical Sciences, University of Copenhagen, Copenhagen, Denmark; The Bartholin Institute, Rigshospitalet, Copenhagen, Denmark

## Abstract

Huntington’s disease (HD) is a neurodegenerative illness, where selective neuronal loss in the brain caused by expression of mutant huntingtin protein leads to motor dysfunction and cognitive decline in addition to peripheral metabolic changes. In this study we confirm our previous observation of impairment of lactate-based hepatic gluconeogenesis in the transgenic HD mouse model R6/2 and determine that the defect manifests very early and progresses in severity with disease development, indicating a potential to explore this defect in a biomarker context. Moreover, R6/2 animals displayed lower blood glucose levels during prolonged fasting compared to wild type animals.

## Introduction

Huntington’s disease (HD) is neurological disease caused by a dominant trinucleotide expansion mutation (CAG) in the huntingtin gene leading to a dominant gain of function of the mutant huntingtin protein (mHTT)^1^. Due to neuronal cell loss in specific brain regions, particularly the striatum, patients experience motor dysfunction, cognitive decline as well as psychiatric symptoms^2^. There is currently no known cure for the disease. Specifically, a CAG repeat length expansion of 39 or more is sufficient to cause HD^1^^,^ 3. The huntingtin gene is globally expressed, and mHTT accumulates over time, forming cellular inclusions^4^^,^ 5. In addition to the neuropathological changes characterizing HD, peripheral cell dysfunction is widespread^6^.

Previously, we have investigated the glucose and lactate metabolism in HD patients and in a transgenic HD mouse model. We observed that the glucose peak normally observed in healthy individuals following cessation of exercise^7^ is not seen in HD patients; an observation which further investigations using the R6/2 HD mouse model showed were likely to be caused by a reduced capacity for lactate-based gluconeogenesis^8^. Gluconeogenesis, *de novo* synthesis of glucose from precursors including lactate, glycerol and amino acids^9^, is particularly important during fasting^10^^,^ 11 as a means to maintain blood glucose levels and is involved in considerable metabolic exchange with both glycogen metabolism^12^ and the citric acid cycle^13^^,^ 14. Thus, hepatic metabolic dysfunction in the form of gluconeogenic impairment could attribute to weight loss seen in the R6/2 model^15^ as well as HD patients^16^^,^ 17 ^,^ 18 ^,^ 19 ^,^ 20. In this study, we further characterize these changes by investigating gluconeogenic capacity longitudinally in the R6/2 mice and the potential impact on blood glucose regulation during fasting.

## Methods

Reagents were purchased from Sigma-Aldrich, unless otherwise specified.

**The R6/2 mouse line**

The R6/2 mouse line is a transgenic HD mouse model expressing exon 1 of the human huntingtin gene^15^ with a ~144 CAG repeat expansion. Breeding was performed using CBA/J x B6 as a background strain. Transgene presence was confirmed in offspring using DNA derived from tail tip samples by PCR amplification using mHTT-specific primers (forward: ATGGCGACCCTGGAAAAGCTGATGAA, reverse: CGGCGGTGGCGGCTGTTG). Following gel electrophoresis, PCR reaction products (approximately 500 bp) were stained with a DNA dye for visual inspection. Wild type (WT) littermates were used as control animals. All animals had free access to water and standard laboratory chow, except during fasting (16-18 h over night), where the mice only had free access to water. The mice were kept at a 12-hour light and dark cycle. Experiments were approved by The Danish Animal Experiments Inspectorate under permit no. 2012-15-2934-00039. All mice were bred at the Department of Experimental Medicine at the Panum Institute, University of Copenhagen. The mice used in this study originated from a colony maintained at Lund University, Lund, Sweden.

**Lactate challenge and fasting blood sugar levels**

Mice were fasted for 16-18 h over night and fasting blood glucose determined by tail tip blood sampling of 10 μl blood, using EDTA-coated capillary tubes (Vitrex, Denmark), which were then transferred to Eppendorf tubes containing 100 μl of perchloric acid for glucose determination (Merck). Subsequently, 4 μl per g body weight of L-lactate solution (0.5 mg lactate/ul sterile H_2_O) was administered by intraperitoneal injection in order to achieve a final dosis of 2 mg/g bodyweight^21^. Following the injection, further blood samples were taken collected at specific time-points over a 2 hour period, after which the mice where returned to the home cages. Supernatant for analysis of glucose and lactate content was obtained by spinning 2 min at 10,000 g, after which samples were stored at -18 °C.

Fasting blood sugar was measured in blood samples taken every 2 hours as described above, beginning at 9 a.m. The animals were housed together in their cages as usual during the experiment.

**Lactate and glucose determination**

Samples were assayed for glucose and lactate content by fluorometric determination of NADP^+^ and NAD^+^ reduction using a Fluoroskan Ascent Reader (ThermoFisher Scientific) with a 355/460 nm filter pair. In both cases, 3.7 μl sample was added to 150 μl assay buffer (glucose assay buffer: 0.2 M Tris, 2 mM MgCl_2_, 0.33 M NADP^+ ^(Roche),3 mM ATP (Roche), 0.9 U/ml glucose-6-phosphate dehydrogenase (Roche), pH 7.5; lactate assay buffer: (0.5 M glycine, 2.5% hydrazine solution (80%), pH 9 with 3 mM NAD^+^ (Roche) and 33 U/mL L-lactate dehydrogenase (Roche)) in a 96-well plate. For lactate, the plate was read after incubating 60 min at room temperature. For glucose, the plate was read twice; once 5 minutes to establish well background fluorescence, and again after addition of hexokinase (Roche) (final concentration 0.7 U/mL) and 30 minutes further incubation. All measurements were performed in duplicate.

**Statistical analysis**

Linear regression for standard curve calculations of glucose and lactate was carried out using DataFit (Oakdale Engineering). T-tests were performed using Graphpad Prism (Graphpad Software Inc.) and area under the curve (AUC) calculated with NCSS. Mixed-design analysis of variance (Split Plot ANOVA) was made with IBM SPSS (IBM). Pairwise comparison analysis for Split Plot ANOVA was adjusted for multiple testing using the Bonferroni correction. All data are shown as mean±standard error of mean (SEM).

## Results

We previously demonstrated abnormal lactate- and glucose metabolism in 10-week-old R6/2 mice^8^, indicating an impairment of the gluconeogenic pathway in the R6/2 mouse liver. In order to characterize the development of these changes, relative to the development of symptoms of HD, we performed a longitudinal study of blood lactate and glucose levels after a lactate challenge in R6/2 mice at 4, 6, 8 and 10 weeks of age (n = WT: 5 males + 6 females, R6/2: 7 males + 5 females). In our laboratory, the R6/2 mice show clear signs of HD at an age of 8 weeks^22^. Figure 1 shows the peak levels of both lactate and glucose in R6/2 relative to WT mice, as well as the AUC, revealing that the lactate clearance appear to be affected in mice as early as 4 weeks of age (AUC, p = 0.01) whereas the post-injection glucose response is significantly reduced from 8 weeks of age.

**Longitudinal lactate challenge indicating gluconeogenic capacity.**
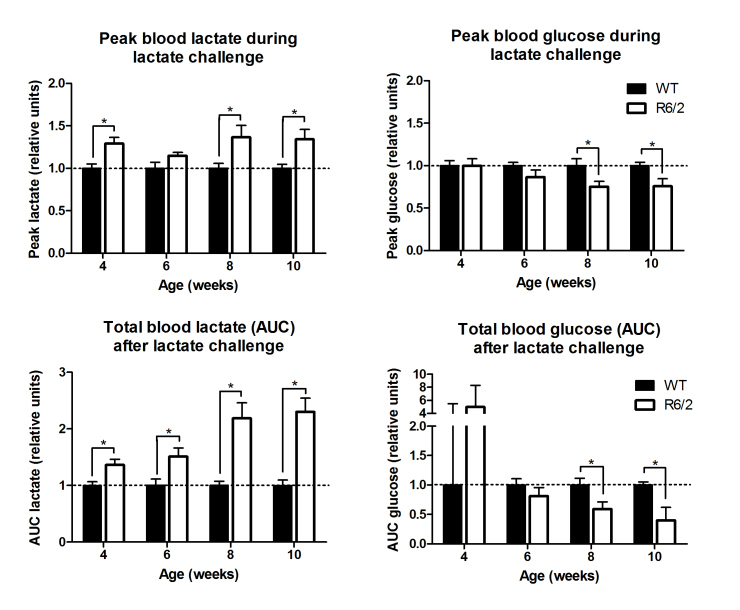
Injected lactate is converted to glucose by the liver through gluconeogenesis. Lactate conversion deteriorates with age in the R6/2 mice compared to control animals as shown by total area under curve (AUC) and peak concentration of blood lactate and glucose after a lactate challenge (WT: 5 males + 6 females, R6/2: 7 males + 5 females). All data is shown relative to the WT mean. Mice were fasted prior to the experiment. P-values < 0.05 are indicated by asterisks above the bars. Groups were compared using a Student’s t-test.

We then measured the blood glucose level during fasting (Figure 2), and found that overall, blood glucose levels significantly differed between 10 week old R6/2 and WT mice during fasting (Split Plot ANOVA, p=0.046, n=10+10 males in each group). Specifically, blood glucose levels were significantly reduced in R6/2 mice compared to WT animals at 8 hours of fasting and at all time-points tested after 12 hours of fasting (p<0.05 for all timepoints, except 14h: p = 0.05). Conversely, R6/2 mice appear to display higher blood glucose levels than WT mice at the initial stages of fasting (2h), however this effect was not significant (p=0.13). Baseline blood glucose did not differ between the groups (WT 3.10±0.32, R6/2 3.03±0.47, p=0.88).

**Fasting blood sugar in R6/2 mice.**
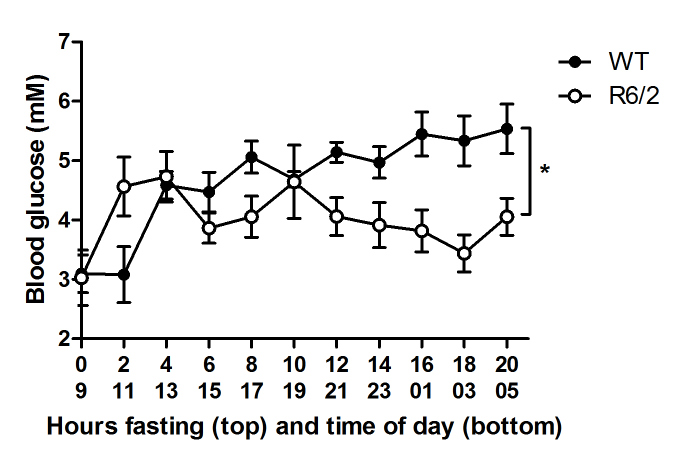
R6/2 mice display lower levels of blood sugar overall during prolonged fasting relative to control animals (Split Plot ANOVA, p=0.046 and n=10 males in each group).

## Discussion

This study shows that hepatic gluconeogenesis in R6/2 mice is affected from an early age and appears to deteriorate with disease progression, causing hypoglycemia during extended fasting.

The hepatic impairment in lactate metabolization in fasted R6/2 mice suggest a direct effect of mHTT on hepatic gluconeogenesis. Since gluconeogenesis also plays a role in glycogen and amino acid metabolism^23^^,^ 24, a decreased gluconeogenic capacity could also contribute to changes in the dynamics of other metabolic pathways in the liver. However, lactate is not exclusively metabolized through hepatic gluconeogenesis, although extrahepatic mechanisms could also contribute. While speculative, this could imply that R6/2 mice might compensate for metabolic changes caused by mHTT early on, but becomes increasingly unable to do so as time passes. Alternatively, - or in addition - the cause of the defect itself may worsen progressively, like e.g. by increased sequestration of regulatory proteins in mHTT inclusions.

Paradoxically, a reduction in fasting blood sugar was only seen for mice specifically tested to investigate blood sugar levels during fasting and not in R6/2 mice fasted overnight in preparation for lactate challenge testing. This could be related to differences in timing of the fasting period as well as the testing conditions. Lactate-challenged mice were fasted over night, while the fasting experiment was initiated in the morning. Mice are nocturnal^25^, and fasting over night thus extends the daytime starvation period, indicating that nighttime fasting could potentially impact fasting blood sugar levels to a larger degree than daytime fasting. However, R6/2 mice show profound disturbances of circadian rhythm^26^^,^ 27, and may thus experience a more pronounced effect of daytime fasting compared to WT animals. Also, samples were collected continuously in the fasting study, while mice preparing for lactate-challenge were undisturbed during the fasting period. Thus, the frequent handling of the animals could have caused a stress reaction in the R6/2 animals manifesting as hypoglycemia. Curiously, we observed a higher blood glucose level in R6/2 mice compared to WT after 2 hours of fasting. This could be caused by an increase in glycogen breakdown, however the activity of the rate-limiting glycogen phosphorylase enzyme is normal in R6/2 liver^8^.

At 4 weeks, very high variance was observed for blood glucose AUC following a lactate challenge compared to all other data points. Since no similar irregularities were seen for peak blood glucose or lactate data following the lactate challenge at this age, it appears that the timing and length of the post-injection period of hepatic glucose production is very variable in young mice, rather than the maximal capacity of glucose output.

In general, we observed relatively low base-line blood glucose levels in both WT and R6/2 mice. However, comparable levels have been reported previously^28^. The R6/2 line is known to display a diabetic phenotype. This includes increases in fasting blood sugar^29^^,^ 30 ^,^ 31 ^,^ 32; however, we have not observed this in our mouse colony, indicating that development of diabetes may be specific to certain R6/2 colonies.

The clear deterioration of lactate-based gluconeogenesis with age and thus disease progression in the R6/2 mouse strain suggests that this parameter may be suitable for use as a peripheral biomarker in this model. This perspective underscores the importance of further characterization of hepatic gluconeogenesis and carbohydrate metabolism in HD patients.
